# Parental Perceptions of Youths’ Desirable Characteristics in Relation to Type of Leisure: A Multinomial Logistic Regression Analysis of Martial-Art-Practicing Youths

**DOI:** 10.3390/ijerph19095725

**Published:** 2022-05-08

**Authors:** Tony Blomqvist Mickelsson

**Affiliations:** Centre for Baltic and Eastern European Studies, Department of Social Work, School of Social Sciences, Södertörn University, 141 89 Huddinge, Sweden; tony.mickelsson.blomqvist@sh.se

**Keywords:** parent, martial arts, masculinity, bullying, health

## Abstract

Parents place their youths in sport with the belief that doing so will produce developmental outcomes. However, it is unclear if parents enroll children in different sports based on different desired characteristics they wish their youth to develop. This paper analyses the link between youths engaged in martial arts (MA) compared to other leisure activities. MA research has indicated the importance of masculinity and gender ideals that suggest that parents hold certain visions when enrolling their youths in MA. For example, one such vision is for their youths to be able to handle themselves in physical encounters. Two research questions guided the study. First, what characteristics do MA parents desire their children to develop? Secondly, how do these desires correspond to MA youths’ actual characteristics? We utilize multinomial logistic regression analysis on nationally representative data from the Netherlands. The results show that MA parents are younger, their youths are of migration background, and the parents value characteristics such as self-control, responsibility, and acting “gender appropriately”. These results correspond to their youths; MA youths are consistently characterized by more masculinity compared to the youths in other groups. The results bear implications for how MA environments must safeguard against potentially harmful and misleading norms.

## 1. Introduction

Parents play an invaluable role in youths’ participation in and experience of sports. An increasing amount of research has been devoted to parental involvement and how this affect youths’ sporting activities, generally making distinctions between levels of involvement [1]. While it is generally agreed that structured leisure contributes towards youths’ development [2], Coakley [3] noted that complex cultural changes have equated this level of involvement with parents’ own moral worth and thus affected parents’ involvement in youth sports. This seems to be a generational shift in parental involvement in youth sports at least in Western contexts. In the Norwegian context, Stefansen et al. [4] noted that parental involvement has increased dramatically across generations and is seen by parents as a way to connect with their youths emotionally and contribute toward youth development. Likewise, in the English context, Wheeler and Green [5] noted that this involvement is nowadays seen as a core pillar of child rearing.

While it is well-established that parents believe sports will contribute toward youths’ development, it is unclear how parents perceive different leisure activities to be linked to different sorts of developmental outcomes. Sport can be seen as a subcategory under the umbrella term leisure. In turn, leisure can, on a spectrum, be unstructured or structured. While the former may involve “passive” and unorganized activities (e.g., hanging out at the youth recreation center), the latter is usually defined by several parameters contingent on organization. As per Mahoney and Stattin’ [2] (pp. 114–115) definition, structured leisure activities are characterized by “… regular participation schedules, rule-guided engagement, direction by one or more adult activity leaders, an emphasis on skill development that is continually increasing in complexity and challenge, activity performance that requires sustained active attention, and clear feedback performance”. It is with this definition of leisure that the paper proceeds.

In this study, I explored a rather uncharted territory that links parental desires about *different* traits to *which* leisure activity their youths are enrolled in. I focused this analysis on one emerging and debatable specific sport in a comparison with other broader categories, namely martial arts (MA). MA is an umbrella term that covers a range of disciplines, and as such, MA is not easily defined. However, a denominator for various MAs is the development and acquisition of fighting skills [6]. Accordingly, MAs can include, for instance, boxing, taekwondo, wrestling, and karate. For the current study sample, the included MAs are all conceived of as compatible with this definition and share similarities in many aspects. One salient aspect is that they are all Olympic sports and thus considered highly competitively oriented.

Moreover, I accomplished this by using nationally representative data (*n* = 2625) from the Netherlands with data on both parents and their youths in two multinomial logistic regression models. These analyses sought to answer the two following questions: (1) how are parental desires about various traits connected to the type of leisure their youths are enrolled in, and (2) how do the parental desires correspond to the attitudes and characteristics of their youths?

Before proceeding to the method, results, and discussion, I will briefly situate sport and MA into the broader debate about sport’s stratified nature along the continuum of social class to understand why cultural values may fluctuate *across* sports. I will proceed to review the literature on who participates in MA and what their own motives are for doing so. Afterwards, I review the literature on parents’ role in MA: what do they believe MA contributes towards? What is specific about MA?

### 1.1. A Broader Contextual Overview: Sport, Social Stratification, and Cultural Values

Social stratification in sport has received a great deal of scholarly attention [7,8,9] and continues to be a topic of interest in contemporary time [10,11]. The consistent findings have been that social markers, such as class, sex, and ethnicity, are strong predictors for individuals’ sporting participation. For instance, socioeconomic status is consistently linked to participation in both frequency and range of activities [12] and across contexts such as Finland [13], England [14], Flanders [15] and the U.S. [16].

Recently, Mutz and Müller [17] analyzed this aspect again in Germany but with the rationale that previous studies had based their designs on binaries of either participating or not participating, thus disregarding the nuanced stratification that may occur *within* sport. The authors analyzed representative data from Germany, showing how participation in different sports is socially stratified along the traditional continuum of social class. Lower-class individuals were consistently underrepresented in general, while higher-class individuals were overrepresented in, for example, racket sports, nature-based sports, and water-based sports. As Mutz and Müller [17] pointed out, although Bourdieu [18] provided arguments for the different associations between social dimensions and different sports, little research has empirically shown what these relationships look like.

Within this social stratifying of sports, parents play a decisive role in initiating and affecting their children’s sport participation. Parents’ critical role is displayed in an abundance of studies showing how parents impact youths’ sporting experiences through their behaviors [19,20]. Similar to how sports are stratified according to social markers [17] and specific cultural values [18], it is plausible that the parents who are embedded within these structures believe that different sports will contribute towards different (or more profound) outcomes for their children. In other words, some parents may enroll their children in certain sports based on cultural preconceptions of what different sports contribute to and, generally, what characteristics they wish their children to develop. Some examples from Bourdieu [18] include working-class members’ preference for physical and masculine sports, such as weightlifting or boxing, whereas higher-class members would value more refined and culturally influenced activities (e.g., theater). While this study is not primarily concerned with the classed pattern of values and sporting participation, it is nevertheless important to illuminate the fact that parents are embedded within these structures that may predispose them to certain beliefs about different sports’ utility.

In this regard, MA has historically occupied a clearly demarcated position within the social strata. As Bourdieu [18] noted, boxing is associated with working-class preferences for physicality and masculinity. In contemporary time, the research on cultural values associated with MA is not entirely straightforward. An abundance of sociological literature hints at the inherent masculinity in MA [21,22,23,24], which would appeal to working-class members and parents’ preference for their children to develop such traits. Other work has found that masculinity and ideas of gender do not function as an organizing principle in MA gyms [25]. As an example of how divides between SES, gender, and type of sport might be becoming more blurred, Stuij [26] found that, on average, high-SES youths tended to participate in MA to a greater extent than low-SES youths.

Important to the paper’s context is also the link between migrant status, SES, and MA. Migration status is, with only minor exceptions, connected to low SES because of factors such as language barriers, labor- and school-related discrimination, and a generally more difficult path towards the higher social strata. In conjunction, and first, the Netherlands is a country with a history of being a colonial regime, notably with former colonies in Indonesia, Suriname, and the Antilles [27] (Oostindie, 2011). Accordingly, the migrant population is reflective of this history. Secondly, it is also a nation that has achieved great success in MA, particularly in kickboxing. A range of famous Dutch kickboxers have gone down in the history books as defining the sport. Notably, many of these are ethnic minorities, such as Surinamese-Dutch kickboxers Remy Bonjasky and Tyrone Spong, Moroccan-Dutch kickboxer Badr Hari, and Turkish-Dutch kickboxer Gökhan Saki, to name a few. Accordingly, there is a rich tradition of Dutch ethnic minorities becoming MA superstars.

Unsurprisingly, MAs are popular choices for ethnic minorities in the Netherlands [28]. The popularity of MA amongst ethnic minorities has also been picked up by Flemish social initiatives that utilize MA as a means to combat social issues [29]. As such, MA in this study’s context is not only influenced by classed patterns and migrant status [28] but it is also a sport in which ethnic minorities have excelled to the level of stardom to a notable extent. One could thus be led to suspect that MA holds a kind of cultural value for ethnic minorities in the Netherlands, similar to what boxing represents for Afro-American populations in the U.S. [30].

### 1.2. Individual Motives and Parental Perceptions of Martial Arts Involvement

Many studies have reported that people partake in MA because of generic and universal reasons across type of sport. These reasons include health [31,32,33] and desire for affiliation and friendship [31,34,35], where it is assumed that MA gyms will satisfy these needs, much like other types of social venues. Other research has shown that individuals believe that MA practice will give them self-confidence [32,36], discipline, and life skills. Again, these are not unique to MA but can be viewed as part of a family of developmental traits to which the sport is assumed to lead [37].

Moreover, the rationales for partaking in MA share commonalities across groups but also contain a degree of diversity. For example, in Stuij [26], the rationales for participation included ideas of “catharsis” [38], meaning youths vented frustration and aggression through MA. This was more prevalent for boys than girls. On the other hand, girls partook mainly because of a perceived need to learn self-defense. This is consistent with previous research showing that MA is associated with a perceived lower likelihood of being a crime victim [39].

In early studies, Twemlow et al. [36] found that motives amongst young adults include a desire for increased self-confidence but, consistent with “catharsis theory”, also as an outlet for aggression. In general, these findings coincide with research that indicates how MA is connected to increased propensity for violence [40,41] and arguments about the need to strongly reconsider if MA is appropriate [42] for youths [43]. For example, in his ethnography of MA practitioners, Green [22] (p. 427) noted that his (male) participants “… discussed the potential of violence in everyday life to an extent I had not witnessed before” and that these narratives seemed to cultivate a culture that encouraged and emphasized violent abilities as a natural part of being “a man”. In a similar vein, Green [44] (p. 383) found that his inclusion into the MA gym was contingent on his ability to display a range of valued characteristics, including “… aggression, competitiveness, athleticism, and lack of fear of physical conflict”. Likewise, in the narratives of his interlocutors, Spencer [23] found that masculinity was sometimes defined as actions that implied physically dominating other men.

Sport, in general, is conceived of as a bastion where men are “taught to be men” [45] and where masculine elements that do not fit in within society can be cultivated and unleashed [46]. In this regard, Green [22] (p. 423) noted that “… this equation of violence with a lower-class brand of masculinity, with fighting providing a path to establish a “masculine reputation,” fits the trend of the larger discipline”. To further add to this, Spencer [23] argued that this proclivity to violence is often part of young males’ upbringing, in which being able to perform violent acts can be a part of a “healthy” masculinity. It is not difficult to see how the sport-specific aspects of MA would satisfy the pursuit of such masculinities.

Reflective of the ambiguity of MA research, however, later reviews have refuted these initial findings [47,48], generally pointing to increased well-being due to MA practice [6]. Still, it is important to highlight that these results have nothing to do with the initial *perceptions* of MA benefits but are results of the MA practice itself or, at least, empirical associations. Taking stock of these perceptions, it is evident that they include common reasons (e.g., health) but also because individuals believe, to various degrees, that MA provides them with unique benefits. This includes the acquisition of physical skills necessary to defend oneself and, to a lesser extent, a potential forum to “release” negative emotions. All in all, these latter perceptions may be connected to ideas of the cultural value of masculinity. On the contrary, the need to acquire self-defense skills do not necessarily indicate that masculinity is a driving cultural value but instead reflective of an increasing neoliberal emphasis on individuals’ responsibility to be responsible for and, in this case, be able to defend themselves.

### 1.3. What Current Parental Beliefs Exist about MA Involvement?

Since parents constitute the main socializing agent for youths’ sporting involvement, it is important to explore what parental perceptions of MA involvement exist. In general, it seems that MA practitioners with strong parental support also have stronger participation motivation [49]. Moreover, several authors reported that parents view MA as a way for their children to learn self-defense and to develop important characteristics such as self-confidence [50,51]. The latter is no surprise, seeing that MA represents an arena dedicated to traits such as discipline, self-control, and self-confidence [52]. 

A recently published doctoral thesis [53] found that parents’ perceptions of MA’s educational value could be traced to three distinct domains of self-regulation: cognitive, affective, and physical. The parents believed that, for instance, MA practice would lead to improved goal setting and academic performance, a more positive mindset, and, importantly, protect against victimization. Again, while goal setting and academic performance generally relate to perceptions of most sport activities, MA as a preventive measure against victimization is clearly specific to MA.

The educational value of MA has also been corroborated elsewhere. In a South Korean sample, it was found that parents enrolled their children in taekwondo because of a belief in its developmental contribution, which could provide their children with tools to enter adulthood successfully [54]. Even during the COVID-19 pandemic, parents have believed in the educational contribution of MA to the extent where they enroll their children in Internet-delivered MA practice [55,56]. The underlying mechanism seems to be that parents perceive MA to contribute toward intra-personal characteristics, such as self-confidence or discipline, that will help their children later in adulthood. Finally, preliminary evidence shows that parents perceive the value of MA differently based on variables such as sex [50], with females viewed as in most need of self-defense training [26]. In general, these results suggest that parental perceptions of MA’s contribution vary according to sex.

In summary, the literature on MA participation indicates several things. First, MA participation is conditioned by generic motivational factors, such as health or enjoyment. However, participation is spurred by an emphasis on the ability to defend oneself in physical encounters. This sort of ability does not develop naturally in other sports. On one hand, MA is frequently conducted as a form of self-defense intervention for women [57]. On the other hand, extensive research has shown that MA environments are usually impregnated with ideals of masculinity [23,58,59,60]. In turn, these masculinity ideals may be a decisive factor when parents enroll their children into MA, which are ideals that may not be as salient in other sports. The ideals are often connected to violence and males’ ability to exert violence [22,44] as a natural part of “being a man”. This link between masculinity and violence is tentatively where MA presents itself as a particularly interesting context.

Regardless of this, MA research is characterized by an emphasis on the role of masculinity and how MA continues to reflect and stimulate these ideals. Generally, the scant literature on parental perception of MA has indicated a certain predisposition toward believing MA will bring about discipline and the ability to defend oneself. These views seem gendered and conditioned by a perception that children need to develop cognitive and physical abilities they can leverage in various contexts.

## 2. Materials and Methods

The study uses the Children of Immigrants Longitudinal Study in Europe (CILS4EU) data. CILS4EU focuses on several markers of well-being and integration into host society, such as social networks, sport and leisure behavior, structural integration (i.e., school and labor market), cultural integration (e.g., language), and psychological integration (e.g., general life satisfaction and emotional belonging). The CILS4EU data include not only migrant children and parents but also include native children and parents; however, due to the emphasis on integration, migrants are intentionally oversampled in the CILS study. The study includes Germany, the Netherlands, Sweden, and England. For the paper’s purpose, we used the data from the Netherlands from the first wave of data collection [61]. These data were collected in 2010/2011 when respondents were around 14 years of age. The reason for this selection is that this is the only wave and country to differentiate between types of leisure. CILS4EU adopted a stratified three-stage sample design. The first stage sampled schools selected by probability proportional to size. The second stage sampled classes within these schools. The third stage sampled students within the classes. In total, 109 schools and 222 classes were sampled in the Netherlands, equally spread out over four socioeconomic strata. Schools within lower levels of this stratum were intentionally oversampled to increase the sample size of migrants. The final sample for this wave was 4363 students. This study used the leisure active youths out of this sample, ultimately consisting of 2625 (*n*). Prior to data collection, the study underwent ethical procedures according to each country’s directives. In the Netherlands, no formal ethical approval had to be sought, but the CILS team nevertheless followed the same ethical procedures as in the other included countries. For this study, the use of the data was further ethically approved by the Swedish Ethical Authority Review.

### 2.1. Demographics

The study controlled for several demographics at both the youth and parent level. For parents, age was coded according to birth year and calculated according to their age at the time of replying to the survey. The youths were restricted in the age range and were born between 1993 to 1997. Due to small cell sizes in 1993 and 1997, the variable was dichotomized. Therefore, younger participants (1993/1994/1995) were coded as the reference group and older (1996/1997) as 1. Sex was coded as male = 0 and female = 1 at both the parent and youth level. Socioeconomic status was assessed by the ISEI index [62]. The ISEI index uses scores on education, income, and occupation. The ISEI index scales occupational groups to maximize the indirect effects of education on income and minimize the direct effect of education on income (net effects). It results in a score on the occupation that should reflect SES. In principle, this ranges from 0–100, where lower values are higher SES and vice versa. We summarized both parents’ scores on the ISEI as a composite score for SES. Migrant background was assessed using one categorized variable where first- and second-generation migrants were coded as 1. The rest (2.5th second-generation migrants up until natives) were coded 0. This variable was only applicable at the youth level since the same categorization did not exist at the parent level.

### 2.2. Dependent Variable

The dependent variable was type of leisure activity, with a focus on MA. Four types of leisure activities were categorized, constituting of team sports (e.g., soccer, ice hockey, handball), individual sports (e.g., swimming, tennis), cultural activities (e.g., singing, theater), and MA (e.g., judo, karate, taekwondo). This was done through two items. First, one item asked whether the youths had any sort of membership in a club. Secondly, the youths indicating that they had a membership were asked what type of club this was. This operationalization was based partially on Bourdieu’s [18] notion of sports and different cultural and classed patterns (e.g., boxing versus theater) but also on research that has shown how different traits (e.g., aggression) differ between different broader categories of sport [63].

### 2.3. Independent Variables

The parental predictors included 10 questions about characteristics that they deemed important for their child to develop. Here, parents were instructed to note the most important characteristics. These characteristics spanned different theoretical constructs. In its empirical form, parents were asked about the following characteristics’ importance: (1) being responsible, (2) working hard to achieve goals, (3) having high degrees of self-control, (4) being generally interested in one’s surroundings, (5) having good manners, (6) having good judgment, (7) being considerate of others, (8) acting gender appropriately, (9) respecting the elderly, and (10) obeying one’s parents. Out of these, “acting gender appropriately” was the most important for the current analysis. The specific item here was the following statement: “*That s/he acts like a boy/girl should*”. Out of these characteristics, parents were asked to indicate the three most important. These were rated on a binary scale, thus indicating whether the trait was indeed one of the most important traits for their child to develop or not. The predictors tested were partially derived from previous literature (including the ones asking about gender roles, self-control, etc.), and others were adopted in an explorative manner. Finally, we included a measure on parents’ levels of tolerance as an additional measure of norms indicative of conservative gender roles and masculinity. This variable was indexed by four items asking about (1) the respondents’ perceptions about cohabiting without being married, (2) divorce, (3) abortion, and (4) having a homosexual orientation. These were rated on a 1–4 scale, where lower points indicated higher agreement. These items were aggregated, and the scale was reversed so that higher points indicated higher agreement. We assumed that being more conservative would also serve as a proxy for masculinity, as it has been shown that conservative values and perceptions about sexual orientations and traditional family structures correlate with individuals’ levels of masculinity [64,65,66,67].

The main predictors for youths’ activity-belonging constituted three variables. First, we constructed the same variable for tolerance as for the parents. Secondly, and importantly, we indexed three items that pertained to masculinity. This included the following statements: (1) “*A man should be ready to use violence to defend his wife and children*”, (2) *A man should be ready to use violence against insults*”, and (3) “*A man should be ready to use violence if someone talks badly about his family*”. These were rated on a scale from 1–5, where lower points indicated stronger agreement. These questions were indexed and reversed so that lower points indicated lower masculinity and vice versa. It is worthwhile noting that this definition of masculinity is very narrow, as it emphasizes scenarios including violence. However, it is also a definition of masculinity that coincides with much of the research showing how sport-specific (violent) elements of MA are connected to ideas of masculinity [22,23,44]. Finally, we assessed the levels of being victimized in school. This variable was constructed by indexing three items. These items included whether the youth during the last month had been (1) scared of other students, (2) teased by other students, and (3) bullied by other students. The items were rated on a scale from 1–4, where lower points indicated more rates of victimization. The items were aggregated and reversed so that higher points indicated higher rates of victimization and vice versa. Importantly, it is worth noting that the operationalization of the indexed items followed the CILS team’s operationalization of masculinity, victimization, and tolerance, see technical report [68].

### 2.4. Analytical Procedure

In the first stage, descriptive statistics were explored amongst both parents and youths. In the second stage, we deployed two multinomial logistical regression models. The first assessed parental predictors of their youth’s involvement in type of activity. The second multinomial model assessed youths’ predictors (masculinity, tolerance, and rates of victimization) on their relation to type of leisure activity.

## 3. Results

We first assessed descriptive statistics, as shown below in Table 1.

A brief takeaway from Table 1 is that MA practitioners seemed to consist of more migrants and report fairly higher levels of masculinity compared to the other groups but also were quite balanced in terms of sex distribution. Subsequently, the first model assessed the parental predictor on choice of youths’ leisure activity. The results are displayed in Table 2.

The odds for being an MA parent were higher if the respondents were comparatively younger and of migration background. Moreover, MA parents had better odds of being club-active themselves except for when compared to cultural activities. MA parents were more likely to select traits of responsibility, self-control, acting gender-appropriately, and respecting the elderly compared to all other groups. Traits that were statistically significant and more likely to be picked in other groups included good manners and being considerate. Secondly, we assessed the youths’ predictors on what type of leisure activity they were enrolled in. The results are displayed in Table 3.

Amongst youths, the odds were higher for being enrolled in MA if respondents were of migrant background (except compared against cultural activities). Additionally, higher rates of masculinity increased the odds for being enrolled in MA compared to the other groups. It was also more likely to be an MA practitioner, as rates of bullying increased compared to team-sport practitioners.

## 4. Discussion

This study set out to understand what characteristics parents perceive as important and how this relates to types of activities their youths are enrolled in with a specific focus on MA. I further attempted to understand whether parents’ ideas about desired characteristics corresponded to youths’ self-reporting on relevant traits.

The main findings were that parents with youths in MA generally value a distinct set of traits: responsibility, self-control, respecting the elderly, and, importantly, acting gender-appropriately. In contrast, the odds were greater of being enrolled in other activities when parents indicated that good manners and being considerate to others were important characteristics for their youths to develop. The first set of characteristics share some denominators. First, to some extent, they emphasize hierarchy (e.g., obey parents and respect the elderly). While all forms of structured leisure activities contain a structure with a leader, this aspect is certainly magnified in MA. The hierarchical structure of MA is reflected in things such as belt levels, where the white belt represents the novice, and the black belt represents the most skilled. The MA trainer is also usually viewed as a role model not only in sport aspects but also in life [50], and research shows that the MA trainer is crucial for the students’ experiences [69]. This may be particularly so given the inherent (physical) risk associated with MA. There is thus a salient power-laden relationship between MA trainers and students.

Secondly, the remaining characteristics entailed inter-personal characteristics that emphasized the individual’s responsibility, self-control, and acting according to the youth’s gender. From a psychosocial perspective, this is consistent with the MA research that emphasizes self-control as a main feature of MA practice [70]. Through disciplined MA practice, individuals learn to control emotions, bodily sensations, and exert “controlled” aggression within a confined space [52,71]. Ultimately, it is expected that this will translate into life skills, as shown by the expectations of MA parents [54]. However, a broader sociological outlook on this matter sheds a different light on the emphasis of these intra-personal traits. Through a different lens, the emphasis on self-control, responsibility, and alike signals a neoliberalist value of MA practice for parents, where the individual is responsible for her own success. This reasoning should be kept in mind juxtaposed with the finding that MA parents consistently downplayed the value of kindness; being considerate to the people in their surrounding was less important compared to all other groups. However, it must be noted that this neoliberal element within the current analysis is only of speculative character. Further research would be needed to tease out whether neoliberalist values are indeed stronger within the MA environment compared to other sports and whether this is salient to the parents and youths themselves.

In the analysis of how parents’ perceptions corresponded to the youths’ levels of masculinity, tolerance, and levels of bullying, the first constituted the silver lining in this study. Whereas tolerance remained an insignificant finding throughout the study, and rates of bullying were only significant compared to team-sport practitioners, MA practice was associated with higher odds of having higher degrees of masculinity compared to all other groups. Interestingly, it is worthwhile to note that, as in most survey research, the sample of parents consisted primarily of mothers. To further add to this, the youth sample was evenly balanced, with an adequate number of female youths present in each leisure activity. It is here important to note that the questions of masculinity emphasized the perceived importance of males adopting conservative and traditional values (e.g., a *man* should defend his family, a *man* should be ready to exert violence, etc.). Although not asked about their own gendered role, the MA sample was consistently associated with higher degrees of masculinity and ideas of acting gender-appropriately compared to all other sorts of leisure activities.

One salient limitation is the cross-sectional nature of the data. Accordingly, it is unclear whether parents’ preferences for strong hierarchal and individualistic values drive them to enrolling youths in MA or whether MA practice functions as the catalyst for such values. Likewise, it is unclear whether MA youths’ masculinity is the result of MA practice or is a reason for why they join MA in the first place. Previous research has shown that MA may attract more aggressive youths although this has not been a consistent finding [72,73]. However, it is clear that there is a strong link between values of gender, masculinity, and MA.

## 5. Conclusions

The current study shows that MA parents value a set of characteristics that emphasizes youths’ self-control, responsibility, obedience, and their drive to succeed while downplaying collective values, such as being considerate to others, acquiring good manners, and more. Importantly, MA parents believe it is more important to act gender-appropriately compared to other activities. These findings correspond to their youths, who consistently display more (conservative) gender-appropriate norms about the male’s societal role. To the best of my knowledge, no study has thus far connected parents’ beliefs about different developmental traits and their links to different sorts of leisure activities. The study therefore contributes to the corpus of literature that has taken an emerging interest in parents’ role in shaping the context of youth sport. Specifically, this study contributes toward preliminarily establishing an empirical link between MA parents and their youths in how their values seemingly align with each other’s. Due to the cross-sectional nature of the data, however, it is impossible to disentangle the causal trajectories. While one might suspect that parents transmit their values to their youths, it is less clear whether MA causes these values or whether these values are responsible for the onset of MA participation. Nonetheless, since youths’ development is contingent on the sporting environment they reside within, it is imperative that we understand the cultures that inhabit specific sports and places [74]. This is an important piece of the analytical puzzle since parents indeed constitute one of the main agents in the onset and continued sport participation of youths. It is also important to be wary of potentially harmful norms that may reside within MA environments and that may spark or exacerbate antisocial behavior [40]. Ironically, this would seem to contradict the inherent educational value that seems to perceptually exist within MA too [54]. It should also be noted that MA is not a homogenous concept or sport. Quite the contrary, the diversity of disciplines that reside under the umbrella term MA are associated with different psychosocial outcomes [72,75,76]. In the current study, the MA group consisted of a range of disciplines; however, the sample size did not allow for a further differentiation in the analysis. Further research would be needed to tease out whether there exist different associations between parental perceptions, youths’ characteristics, and different MAs. It may also be the case that different MAs hold different values for different ethnic groups, such as in the case of Asian parents and taekwondo, for instance. There is thus a need to make further fine-grained analyses that take into account the diversity of MA and how this relates to different groups. As noted by Meyer and Bittmann [32] in their study on Japanese and German karatekas, while many similarities exist in participation motives, their results also indicate that cultural differences play a factor.

## Figures and Tables

**Table 1:** Participant descriptors based on youth and parent data.

	Martial Arts (*n* = 165)	Team Sport (*n* = 1427)	Individual Sports (*n* = 582)	Cultural Activities (*n* = 451)
Age (younger/older)	59/50 (*n*)	492/653 (*n*)	191/275 (*n*)	145/225 (*n*)
Sex (male/female)	69/42 (*n*)	744/413 (*n*)	164/306 (*n*)	90/284 (*n*)
Migrant (native/migrant)	83/28 (*n*)	1058/103 (*n*)	450/20 (*n*)	325/49 (*n*)
SES	90.81 (30.43)	97.38 (33.37)	96 (33.11)	97.38 (34.43)
Masculinity	6.33 (2.92)	5.4 (2.51)	4.51 (2.27)	4.24 (2.38)
Bullied	1.02 (1.43)	0.77 (1.31)	0.96 (1.44)	1.05 (1.51)
Tolerance	6.53 (3.04)	6.74 (2.68)	7.09 (2.44)	7.05 (2.6)
**Parents**				
Parent age	44 (5.09)	46 (4.38)	46 (4.31)	47 (4.81)
Parent sex (male/female)	24/75 (*n*)	245/805 (*n*)	85/352 (*n*)	72/273 (*n*)

**Table 2:** Predicting group membership by demographics, desirable traits and parents’ levels of tolerance with martial art as reference group.

Reference: Martial Arts	Team Sport	Individual Sport	Cultural Activities
	*OR* (Std. Err)	*OR* (Std. Err)	*OR* (Std. Err)
Sex	1.72 (0.1) ***	1.87 (0.12) ***	2.11 (0.12) ***
Age	0.92 (0.00) ***	0.91 (0.00) ***	0.89 (0.00) ***
Migrant	0.31 (0.01) ***	0.14 (0.01) ***	0.46 (0.01) ***
SES	1.01 (0.00)	1.01 (0.01)	1.01 (0.01)
Responsible	0.74 (0.08) ***	0.89 (0.09) ***	0.59 (0.1) ***
Tries hard to succeed	0.73 (0.09) **	0.97 (0.09)	0.71 (0.1) ***
Self-control	0.69 (0.15) ***	0.62 (0.09) ***	0.46 (0.04) ***
Interested	0.7 (0.08) **	0.94 (0.1)	0.94 (0.1)
Good manners	1.49 (0.08) ***	1.61 (0.09) ***	1.37 (0.1) ***
Good judgement	1.02 (0.08)	0.98 (0.09)	1.14 (0.1)
Considerate	1.53 (0.08) ***	1.63 (0.09) ***	1.92 (0.09) ***
Act gender appropriately	0.56 (12) ***	0.59 (0.09) ***	0.41 (0.03) ***
Respect the elderly	0.66 (0.08) ***	0.67 (0.1) ***	0.66 (0.1) ***
Obey parents	1 (0.11)	0.96 (0.08)	0.76 (0.05) ***
Tolerance	1.02 (0.06)	1.01 (0.06)	0.97 (0.06)
Constant	7.39 ***	1.35 ***	1.48 ***

Note. *p*-value. ** *p* < 0.01, *** *p* < 0.001. Nagelkerke R^^2^ = 0.03, Cox and Snell’s R^^2^ = 0.03.

**Table 3:** Youths’ demographics, masculinity, tolerance, and levels of being bullied.

Reference: Martial Arts	Cultural Activities	Individual Sport	Team Sports
	*OR* (Std. Err)	*OR* (Std. Err)	*OR* (Std. Err)
Constant	15.88 (0.77)	36.18 (0.76)	67.39 (0.71)
Sex	2.31 ** (0.33)	1.53 (0.32)	0.59 (0.3)
Age	1.57 (0.3)	1.66 (0.28)	1.64 (0.27)
Migrant	0.79 (0.46)	0.17 *** (0.56)	0.42 * (0.43)
SES	1 (0.004)	1 (0.004)	1.01 (0.004)
Masculinity	0.74 *** (0.07)	0.73 *** (0.06)	0.81 ** (0.06)
Bullied	0.95 (0.09)	0.9 (0.09)	0.83 ** (0.08)
Tolerance	0.93 (0.06)	0.91 (0.06)	0.94 (0.05)

Note: * *p* < 0.1; ** *p* < 0.05; *** *p* <0.01. Nagelkerke R^^2^ = 0.66, Cox and Snell’s R^^2^ = 0.74.

## Data Availability

All data are available from the CILS team.
